# Two decades of cholera in Dhaka, Bangladesh: Epidemiology, seasonal patterns, and environmental risk, insights from 2004–2024

**DOI:** 10.1371/journal.pntd.0014620

**Published:** 2026-08-12

**Authors:** Mohammad Ashraful Amin, Md. Asif Ahsan, Zahid Hasan Khan, Md Taufiqul Islam, Ali Shafqat Akanda, Nasif Hossain, A. S. G. Faruque, Firdausi Qadri, Ashraful Islam Khan

**Affiliations:** 1 Infectious Diseases Division, International Centre for Diarrhoeal Disease Research, Bangladesh (icddr, b), Dhaka, Bangladesh; 2 Department of Civil and Environmental Engineering, University of Rhode Island, Kingston, Rhode Island, United States of America; 3 Division of Infectious Diseases and International Health, School of Medicine, University of Virginia, Charlottesville, Virginia, United States of America; 4 Nutrition Research Division, International Centre for Diarrhoeal Disease Research, Bangladesh (icddr, b), Dhaka, Bangladesh; National Institute for Research in Bacterial Infections, INDIA

## Abstract

Cholera remains a critical public health challenge in Dhaka, Bangladesh, influenced by complex environmental and demographic drivers. This study analyzed around 20 years of 2% surveillance data (2004–2024) from icddr,b (n = 37,361) to investigate long-term trends and climatic associations using distributed lag non-linear models (DLNMs). Climate data (temperature, rainfall, humidity) were obtained from NASA’s Earth Data archives. Findings reveal a significant demographic shift, with adults (>18 years) accounting for 65% of the 6,141 confirmed cholera cases. Seasonality was pronounced; 48% of cases occurred during the pre-monsoon period. While incidence peaked between 2004 and 2008, a recent resurgence was noted from 2019 to 2024. Environmental analysis showed a positive association between temperature and cholera risk, with relative risk (RR) rising from 0.75 at 20°C to 1.10 at 40°C. Precipitation emerged as a major driver, with risk peaking at 150 mm (RR = 1.5), high humidity (90%) also correlated with increased risk (RR = 1.10). The high burden among adults necessitates age-inclusive interventions, including targeted vaccination and WASH measures. Integrating climate data into early warning systems and expanding surveillance to informal settlements are vital for future outbreak prediction and control.

## Author summary

In this study, we examined how cholera has changed over the past 20 years in Dhaka, Bangladesh, focusing on who is most affected, when outbreaks occur, and how environmental conditions influence disease risk. By analyzing long-term hospital surveillance data alongside climate information, we found that cholera is no longer focused among young children, but now affects adults more frequently. We also observed clear seasonal patterns, with the highest number of cases occurring before the monsoon season. Our findings show that environmental factors play an important role in cholera transmission. Higher temperatures, increased rainfall, and high humidity were all linked to greater risk, often with a delay of several weeks. These conditions likely create favorable environments for the bacteria to grow and spread through contaminated water. This work highlights the need to adapt public health strategies to changing disease patterns. Interventions should include all age groups, not just children, and should be timed according to seasonal and climate trends. By combining disease surveillance with environmental monitoring, health systems can better predict outbreaks and take early action to reduce the impact of cholera in vulnerable urban populations.

## Introduction

Cholera remains a major global public health challenge, with recurrent outbreaks reported across sub-Saharan Africa, South Asia, the Middle East, and other endemic regions [1]. In recent years, large-scale outbreaks have been driven by a combination of climate variability, population displacement, inadequate water, sanitation and hygiene (WASH) infrastructure, and fragile health systems, highlighting the need for strengthened surveillance and climate-informed preparedness strategies [2–4]. Despite global efforts to control and prevent cholera, the disease continues to erupt periodically in Bangladesh, including its capital, Dhaka, a cascade effect of environmental-demographic-infrastructure [5,6]. Therefore, understanding the dynamics of cholera over time is crucial for effective control.

Earlier studies have suggested that attack rates of cholera are variable between age strata, with specific sub-populations (e.g., young children and elderly people) at risk [7,8]. There is limited information on the accurate description of age-specific trends in cholera incidence across time spans. Moreover, to our knowledge no study focused on the long-term influence of seasonally varying weather-climate conditions on cholera case counts. Seasonality is another well-described feature of cholera epidemiology, in particular where there are distinct monsoon and dry periods as seen in Dhaka. Cholera seasons are defined as periods of increased incidence, but seasonality across an entire record should be examined in detail for enhanced preparedness and response. Environmental factors such as temperature and precipitation also play a role in the spread of cholera [9]. Changes in temperature may influence the growth of bacteria in water bodies, and rainfall can determine the level of contamination of water and therefore the extent of exposure of the population [10]. In fact, a thorough understanding of these relationships between environment and disease is crucial for making accurate predictions and setting up early warning systems for cholera outbreaks.

There have been several studies on the relationship between urban cholera and climatic factors in Bangladesh. For example, Hashizume et al. [11] analyzed the climatic factors of cholera seasonality in Dhaka by using time-series models but within a short period of time (1996–2002). Similarly, Imai et al. [12] improved the methodological approaches to relate meteorological and infectious disease data using icddr,b data; however, the application was not extended over a long period of time or for different age groups. Research carried out in Matlab, Bangladesh, by Ali et al. [13] and Islam et al. [14] investigated the relationship between climate and cholera in rural areas, which differed from the urban transmission patterns of cholera in Dhaka. Recent studies, such as those carried out by Chao et al. [15] and Hossain et al. [16] using the GEMS dataset, investigated the seasonality of diarrheal diseases and climatic factors in Mirzapur and other low- and middle-income countries; however, the studies were restricted to children under five years of age, which did not specifically target cholera.

The aim of this study was to characterize two decades of laboratory-confirmed cholera epidemiology in Dhaka, Bangladesh, and evaluate the association between climatic factors and cholera occurrence from 2004 to 2024. Specifically, this study addressed the following research questions: (1) How have demographic and temporal patterns of confirmed cholera cases changed over the study period (2) What seasonal patterns characterize cholera upsurge in Dhaka and (3) What are the short-term and delayed associations between climatic variables and cholera incidence. The findings of this study are expected to provide critical information for public health planning, resource allocation, and the development of interventions to mitigate the cholera situation in Dhaka and similar cities.

## Methods

### Ethics statement

The study protocol which approved obtaining the verbal consent from the surveillance patients, was reviewed and approved (protocol # 1992–011) by the Institutional Review Board (IRB) of icddr,b. The surveillance programmed is a regular research activity, monitor the diarrhoea disease pattern, the finding of all data was preserved also for the future use. As this was a retrospective analysis of anonymized surveillance data, no additional consent or assent procedures were required beyond those obtained during surveillance enrollment. However, during enrollment we obtained verbal informed consent from the participants or their parents or legal guardians before enrolling them in the study. Confidentiality and ethical conduct of data were ensured to participants, while consent on usage of anonymized data for research and publication purposes was also included.

### Study design

This observational study was performed using retrospectively analyzed data from the 2% systematic surveillance system on patients admitted to Dhaka Hospital of Icddr,b from 2004 to 2024. The study population included patients admitted with diarrheal diseases over a period of twenty years. An analysis of long-term trends in relation to different age groups within cholera incidence, seasonal fluctuations, and environmental factors such as temperature and rainfall.

### Surveillance system

Frequently, about 200,000 patients with diarrheal diseases visit the Dhaka Hospital of icddr,b yearly. The hospital has a Diarrheal Disease Surveillance System (DDSS), which collects information from every fiftieth patient with 2% systematic sampling technique [17]. During routine surveillance, stool or rectal swab specimens were collected from enrolled participants and tested using standard microbiological procedures for enteric pathogens, including *Vibrio cholerae*. The surveillance data consist of clinical presentations, demographic details and stool sample testing results. DDSS served as the data source for the current retrospective analysis and that all demographic, clinical, and laboratory data used in this study were extracted from this established surveillance platform.

### Identification of pathogens

Those patients who were under surveillance provided stool or rectal swab samples, and they were examined by microbiological methods [18]. *Vibrio cholerae* was isolated and identified by standard procedures that include enrichment in alkaline peptone water and culture on Tellurite Taurocholate Gelatin Agar (TTGA) [19]. These methods were reliable and consistent for pathogen detection during the whole surveillance duration. Microbiological testing was conducted routinely as part of the hospital surveillance system and not specifically for the present study. The current study retrospectively analyzed laboratory-confirmed results generated through routine surveillance activities.

### Clinical data collection

Laboratory-confirmed pathogen identification results, including *Vibrio cholerae* culture results, were extracted from the surveillance database. Data from the 20-year period of demographic variables (age, sex, residence), laboratory-confirmed cholera status, admission date, and surveillance variables were extracted. The trends refer to temporal, demographic, and seasonal trends in laboratory-confirmed cholera cases. As a part of DDSS, clinical data was collected that included patient demographics (age, sex, residence location).

### Climate data collection

The climate data on daily temperature, rainfall, humidity and wind speed measurements was sourced from the freely accessible digitized repository of NASA’s Earth Science Data Systems (Giovanni). The data comprised the period from 2004 to 2024 and were spatiotemporally matched to patients admitted in Dhaka and its surroundings. Daily climate observations obtained from NASA Earth Science Data Systems were aggregated into monthly averages (temperature and humidity) and monthly cumulative totals (rainfall). These monthly environmental measurements were then matched with monthly counts of laboratory-confirmed cholera cases according to admission date.

### Data cleaning and sample size

Eligible participants were patients enrolled through the icddr,b DDSS between 2004 and 2024 who presented with diarrhoeal illness, were included through the 2% systematic surveillance sampling approach, who had proper available demographic, clinical, and laboratory information. Records were excluded if they lacked essential demographic, clinical, laboratory, or date information required for analysis. Additionally, participants residing outside Dhaka district were excluded because climate exposure data were assigned based on Dhaka-specific environmental measurements. To ensure the accuracy and relevance of the analysis, a data cleaning process was performed prior to statistical evaluation. At first, 3,988 participants were not considered as they lacked some necessary data on the clinical, demographic or laboratory side which might lead to improper results. In addition to that, 17,750 participants were excluded because the climate exposure data were assigned based on Dhaka district-specific environmental measurements and those participants were residing outside Dhaka district. This was used as a criterion for consistency in geographical locations and also for the purpose of reducing potential confounding factors that may arise from differences observed in environmental exposures or healthcare access with respect to different regions. After applying these exclusion criteria described before, a total of 37,361 enrolled participants were included in the final analytic dataset for the descriptive analysis of the surveillance population. Laboratory-confirmed cholera cases (culture-positive *Vibrio cholerae*; n = 6,141) constituted the primary analytic population for epidemiological, seasonal, and climate-association analyses.

### Statistical analysis

AWD all enrolled cases diarrhoeal surveillance patients (n = 37,361), used for describing overall diarrhoeal burden. Cholera cases were culture-confirmed *Vibrio cholerae*-positive patients (n = 6,141), used as the primary outcome for seasonal and climate association analyses. The created dataset formed basic input for all types of the descriptive statistics, regression analyses and other inferential approaches envisaged in this survey. Descriptive statistics were used to summarize the counts of acute watery diarrhea (AWD) and cholera cases, as well as environmental variables, across the defined study periods. For all continuous variables, measures including the mean, standard deviation (SD), minimum, and maximum values were calculated. The data were stratified by age group, gender, year group, and season to explore demographic and temporal patterns in AWD and cholera prevalence. A comparison was conducted between the overall cholera-positive population and children aged 0–5 years to evaluate age-specific epidemiological patterns. Association between environmental data and disease trends was investigated. Monthly cholera cases was evaluated in relation to key climate indicators, including maximum temperature, minimum temperature, relative humidity, and rainfall, over the study period (2004–2024). Linear regression models used for exploratory assessment of correlations between monthly cholera counts and individual climatic variables Statistical significance was determined using a p-value threshold of <0.05.

The dependent variable for regression and DLNM analyses was the monthly count of confirmed cholera cases, while independent variables included climatic exposures: maximum temperature, minimum temperature, rainfall, and relative humidity. Climate analyses were performed only using confirmed cholera cases. Distributed lag nonlinear models (DLNMs) was used as the primary analytical approach to evaluate nonlinear exposure-response relationships and delayed effects of climate variables. These models are well-suited for exploring the delayed and potentially cumulative effects of climate variables on health outcomes such as infectious disease incidence. DLNMs were used to estimate the lagged effects of each environmental variable over 0–25 days [20]. We selected a maximum lag of 25 days to capture both short incubation effects and longer ecological/operational delays in the exposure–disease pathway (environmental proliferation, contamination and case ascertainment). The chosen lag window is consistent with the observed cumulative RR peaks at 20–30 days in exploratory analyses and with prior studies showing multi-week delayed environmental effects on diarrhoeal outbreaks [21–23]. Cumulative relative risk estimates were calculated to capture the total effect of prolonged exposure to each climatic variable across the lag structure. We generated three-dimensional (3D) and contour plots to visualize the complex, nonlinear relationships and lag structures between climate variables and cholera risk. Reference lines (RR = 1.0) were included to denote the baseline risk, with values above 1.0 indicating elevated risk. These visualizations aided in interpreting the magnitude and timing of environmental effects on cholera transmission. All statistical analyses were conducted in R (version 4.3.2), and figures were generated using the ggplot2 and dlnm packages [24].

## Result

For Acute Watery Diarrhoea (AWD), a total of 37,361 cases were enrolled, with 18,013 (48%) of cases in the 0–5 years age group, 3023 (8%) in the 5–18 years group, and 16,325 (44%) in individuals over 18 years. The highest number of cases occurred during 2019–2024 (28%), followed by 2014–2018 (25%), 2009–2013 (25%), and 2004–2008 (22%). Pre-monsoon was the most affected season (41%), especially among adults (>18 years: 45%), followed by the monsoon (16%), post-monsoon (17%), and other seasons (26%). Males accounted for a higher proportion of cases (58%) across all age groups compared to females (42%) (S1 Table).

For cholera, out of 6,141 cases reported positive in Dhaka from 2004 to 2024, the majority (65%) were adults over 18 years. Children aged 0–4 years accounted for 17%, and those aged 5–18 years for 18%. The highest proportion of cases occurred during 2004–2008 (36%), followed by 2009–2013 (25%), 2019–2024 (22%), and 2014–2018 (17%). Pre-monsoon season saw the most cases (48%) across all age groups, followed by post-monsoon (22%), monsoon (16%), and other seasons (14%). Males were more affected (58%) than females (42%) across all age categories (Table 1).

**Table 1:** Distribution of cholera-positive cases by age group, year, season, and gender in Dhaka (2004–2024).

Characteristic	Overall, N = 6,141	0-4 years, N = 1,017	5- 17 years, N = 1,116	>18 years, N = 4,008	p-value
**Cholera**					
Positive	6,141 (100%)	1,017 (100%)	1,116 (100%)	4,008 (100%)	
**Year group**					
2004-2008	2,186 (36%)	409 (40%)	489 (44%)	1,288 (32%)	<0.001
2009-2013	1,546 (25%)	288 (28%)	276 (25%)	982 (25%)	
2014-2018	1,061 (17%)	129 (13%)	153 (14%)	779 (19%)	
2019-2024	1,348 (22%)	191 (19%)	198 (18%)	959 (24%)	
**Season**					
Monsoon	1,000 (16%)	168 (17%)	206 (18%)	626 (16%)	0.037
Other	847 (14%)	119 (12%)	146 (13%)	582 (15%)	
Post-monsoon	1,363 (22%)	216 (21%)	258 (23%)	889 (22%)	
Pre-monsoon	2,931 (48%)	514 (51%)	506 (45%)	1,911 (48%)	
**Gender**					
Female	2,559 (42%)	440 (43%)	446 (40%)	1,673 (42%)	0.3
Male	3,582 (58%)	577 (57%)	670 (60%)	2,335 (58%)	

Between 2004 and 2024 in Dhaka, acute watery diarrhea (AWD) cases ranged from 1,030–2,422 annually, with the highest variability observed during 2019–2024. On the other hand, cholera cases showed a declining trend, with the highest average in 2004–2008 and the lowest in 2014–2018. Environmental conditions remained relatively stable across the study periods. Maximum temperatures averaged around 30.6°C, while minimum temperatures averaged 21.3°C. Relative humidity remained high (mean~75%), and precipitation levels were generally low, with occasional peaks, especially in the earlier years (S1 Fig) (Table 2).

**Table 2:** Acute watery diarrhea, cholera cases, and environmental variables across study periods in Dhaka (2004–2024).

Characteristic	2004-2024	2004-2008	2009-2013	2014-2018	2019-2024
Acute watery Diarrhea cases					
Minimum	1,030	1,583	1,563	1,661	1,030
Maximum	2,422	1,783	2,054	2,082	2,422
Mean	1,779	1,666	1,866	1,872	1,723
SD	340	96	217	157	605
Cholera					
Minimum	71	280	199	131	71
Maximum	500	500	455	319	447
Mean	292	437	309	212	225
SD	141	92	109	69	162
Temperature maximum (°C)					
Minimum	18.0	19.4	19.6	18.9	18.0
Maximum	43.3	40.7	42.0	42.5	43.3
Mean	30.6	30.5	31.0	30.5	30.3
SD	3.9	3.9	3.8	3.5	4.1
Temperature minimum (°C)					
Minimum	3.4	7.6	3.4	6.5	7.5
Maximum	28.3	28.1	28.0	28.3	28.2
Mean	21.3	21.2	21.2	21.5	21.3
SD	5.5	5.4	5.6	5.3	5.6
Relative humidity (%)					
Minimum	26	26	27	29	32
Maximum	96	96	95	96	96
Mean	75	75	73	76	77
SD	15	15	16	15	14
Precipitation (mm)					
Minimum	0	0	0	0	0
Maximum	167	167	101	140	145
Mean	6	6	5	7	7
SD	12	11	8	14	12

From 2004 to 2024, seasonal variation in environmental parameters in Dhaka showed distinct patterns. The highest maximum temperatures were recorded during the pre-monsoon season (mean: 34.03°C), while the lowest occurred in the “Other” or the winter season (mean: 26.72°C). Minimum temperatures followed a similar trend, being highest in the monsoon (mean: 26.1°C) and lowest in the “Other” season (mean: 14.5°C). Relative humidity peaked during the monsoon (mean: 89%) and the post-monsoon period (mean: 86%), and was lowest in the drier “Other” and pre-monsoon seasons (both 69%). Precipitation was highest in the monsoon (mean: 12.2 mm), followed by post-monsoon (7.9 mm) and pre-monsoon (7.7 mm), while the “Other” season had minimal rainfall (mean: 0.7 mm) (S2 Table).

Several years, such as 2009, 2010, 2012, 2014, 2015, 2020, and 2022, show a statistically significant positive correlation (p < 0.05) with maximum temperature, (mean range 30.3-31°C), suggesting that higher temperatures are associated with increased cholera cases. However, this trend is not consistent across all years, as many years show no significant correlation (p > 0.05), and a few even show negative associations (S2 Fig). The yearly relationship between minimum temperature and monthly cholera cases from 2004 to 2024 revealed statistically significant positive correlations (p < 0.05) for a majority of the years, including 2004, 2005, 2007, 2008, 2011, 2013, 2014, 2016, 2020, and 2023, show (mean range 21.2-21.3°C), indicating that higher minimum temperatures are generally associated with increased cholera incidence (S3 Fig).

**Fig 1:**
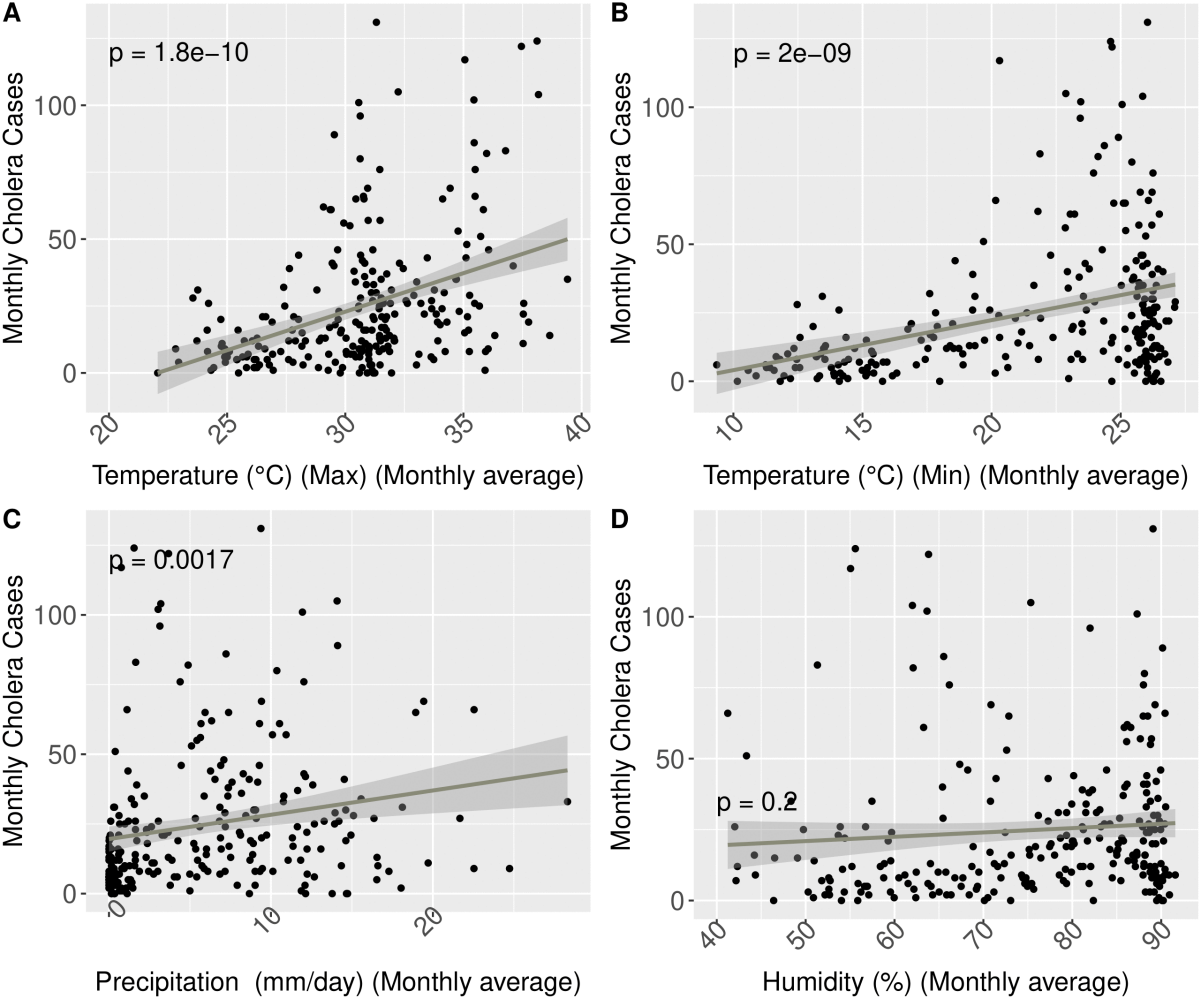
Association between monthly cholera cases and climatic variables from 2004–2024. (A) Average monthly maximum temperature (°C): A strong positive correlation is observed between monthly cholera cases and maximum temperature with a highly significant p-value (p = 1.8 × 10^−10^), (B)) Average monthly minimum Temperature (°C): An even stronger and highly significant positive correlation is evident between cholera cases and minimum temperature (p = 2 × 10^−09^), (C) Rainfall (mm/month): Rainfall showed statistically significant positive correlation with cholera cases (p = 0.0017), (D) Relative humidity (%): had no association was seen between relative humidity and monthly cholera cases (p = 0.2).

For humidity, in the majority of years, the relationship is not statistically significant (p > 0.05) expect 2004, 2007, 2013, and 2022 years, indicating no consistent pattern between humidity and cholera cases over time (S4 Fig). However, for the years (2004, 2007, 2013, 2018, 2021, and 2023) statistically significant correlations with precipitation were observed (p < 0.05), indicating that increased rainfall may be associated with higher cholera incidence during those years (S5 Fig). However, many other years do not show significant trends, reflecting inconsistent patterns over time. Besides, we found a significant correlation of monthly cholera cases with the average maximum temperature (p = 1.8 × 10^−10^), minimum temperature (p = 2 × 10^−09^), and rainfall (p = 0.0017) (Fig 1). Overall, rainfall appears to have a moderate influence on cholera outbreaks, with effects varying notably across different years.

**Fig 2:**
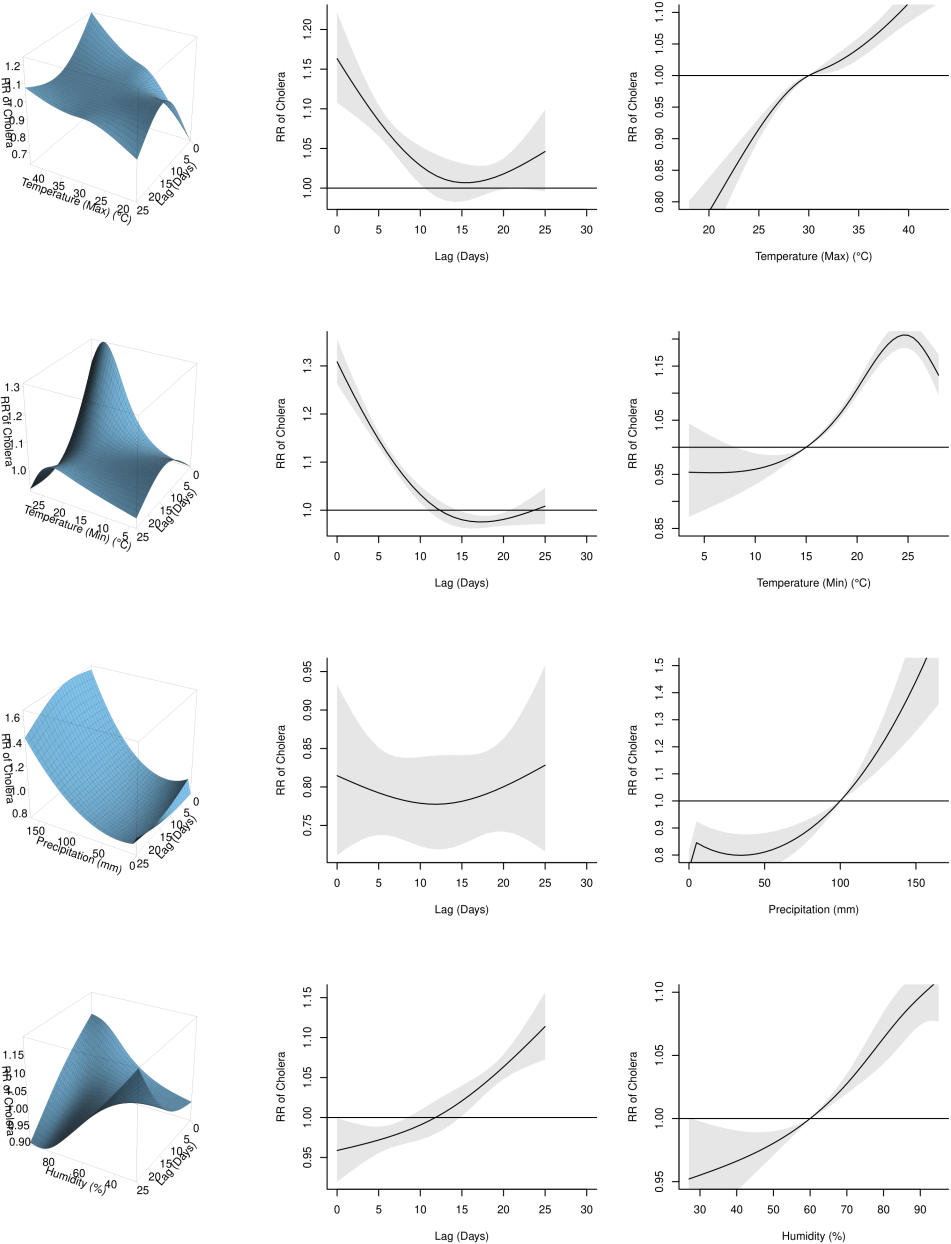
Effects of temperature, precipitation, and humidity on cholera risk over time using distributed lag non-linear models (DLNM).

The distributed lag non-linear model DLNM provided the results for the environmental or weather conditions, like temperature in degrees Celsius, rainfall, and humidity in percent. We showed what the model produced using two and three-dimensional graphs, which showed the relative risk of cholera, changed depending on the amount of exposure and how many days after that exposure. With the temperature, the RR went up consistently from 0.75 at 20°C to 1.10 at 40°C; 30°C was the reference point (RR = 1) meaning that a higher greatest temperature and the risk of cholera have a positively, steadily connected relationship. The lowest temperature did the same, with RR rising from 0.95 at 4°C to 1.20 at 25°C, with 15°C as the reference; this means that the risk of cholera goes up with both the maximum and minimum temperatures (Fig 2).

Concerning rainfall, the RR was at its lowest at 50 mm (RR = 0.8) then rose to the reference level at 100 mm (RR = 1) and reached its highest at 150 mm (RR = 1.5). This shows that large amounts of rainfall might directly raise the risk of cholera. Regarding humidity, lower amounts 30–55% were connected to a lower risk of cholera (RR = 0.95–0.99) whereas higher amounts 70–90% were connected to a greater risk, reaching a highest RR of 1.10 at 90% humidity. These effects were clearest when the delay was longer 20–25 days meaning that a high humidity has a delayed effect on how cholera spreads (Fig 2). A sensitivity analysis checked how well the DLNM held up by changing the longest delay 7–28 days, flexible the exposure was 2–5 df and count distribution was used quasi-Poisson versus negative binomial. The results were the same across the models, showing similar cumulative RRs, overlapping confidence intervals, and steady connections. Small differences in the effects did not change the overall trends, which confirms the dependability and robustness of the DLNM results (S6 Fig).

## Discussion

The analysis gives a complete understanding of the cholera and AWD trends in Dhaka during the last two decades (2004–2024) including the estimation of age-specific patterns of morbidity, seasonality, and environmental factors. The finding has important implications for cholera prevention and control strategies. The demographic analysis revealed that individuals aged more than 18 years were the most cholera positive. Seasonality in terms of very distinct waves was observed, with the pre-monsoon season having almost all-time highest percentage number of cholera cases. The DLNM analysis further helped in understanding the lag response relationships for exposure and the effect of environmental factors on the risk of cholera.

However, each of them has different mechanisms in the seasonal context. The risk of cholera is also increased by heavy rainfall during the monsoon and post, monsoon seasons, which can be attributed to water contamination, sanitation system overflow. The effect of high humidity was moderate and delayed, and it might have increased the survival of the bacteria in the environment, but it depends on the other context like water quality, salinity, and the presence of suitable aquatic reservoirs [25]. The results obtained from this study highlight the significance of seasonally adjusted strategies and early warning systems that consider the individual effects of temperature, rainfall, and humidity on the occurrence of cholera. It is biologically valid considering the incubation period of *Vibrio cholerae* and the time lag between exposure and the peak of the outbreak.

The predominance of cholera among adults’ contrasts with earlier reports emphasizing childhood vulnerability. This shift may reflect changing population immunity, vaccination exposure, healthcare-seeking patterns, or urban transmission dynamics. Such results are inconsistent with previous studies where children, especially those under five, were considered the most vulnerable group [26,27]. Nevertheless, the change in age distribution in Dhaka corresponds to the latest changes in the epidemiology of urban cholera, where the increased immunity of children due to past infection or vaccinations may explain why adults are now more affected [28,29]. Furthermore, the observation of cholera cases has generally gone down over the years from 2004 to 2018, which was then followed by a moderate resurgence from 2019 to 2024, reflecting the effects of water, sanitation, and hygiene (WASH) interventions, changes in health, seeking behavior, or surveillance sensitivity that have been offset by epidemic outbreaks that are influenced by climatic extremes. Declining trends followed by localized resurgences have also been reported in other cholera, endemic regions such as Haiti and the Democratic Republic of Congo, where despite the constant trickle of outbreaks, control efforts are ongoing [30–32].

Seasonality continues to be a foremost characteristic of cholera in Dhaka, showing a clear surge in the pre-monsoon season for all ages. This data is consistent with studies done in India, Nigeria, and Yemen, which reported cholera outbreaks mostly occurring during the seasonal transition between dry and wet periods [33–35]. The pre monsoon outbreak can be explained that *Vibrio cholerae* is maintained in environmental reservoirs and that favorable environmental conditions, including higher temperatures, may increase bacterial persistence, growth, and concentration, thereby elevating the risk of human exposure and disease transmission [36]. We also observed a smaller peak along the post monsoon phase that could be explained by leftover water contamination and the bacterial survival and growth and conducive conditions in the area as it was previously reported [37,38]. While rainfall had a statistically significant association with monthly cholera cases, the influence of rain was modulated strongly by the season. Rainfall before and after the monsoon seemed to have a more significant impact on initiating cholera transmission than heavy rains during the monsoon maximum, most likely reflecting the differences in water contamination routes and dilution effects. Results from a recent research on diarrheal outbreaks in Dhaka provide additional support for these environmental connections. The study revealed that unusually high temperatures, high humidity, and heavy rainfalls directly preceded the outbreaks of cholera and other enteric infections, with *V. cholerae* being highly associated with clinical severity and water source exposure [39]. These seasonal patterns are crucial for designing anticipatory public health responses, including pre-positioning of oral rehydration supplies, water purification materials, and community awareness campaigns.

The distributed lag non, linear model (DLNM) analysis has highlighted the importance of environmental factors in the occurrence of cholera. The delayed nature of this effect can be explained by elevated temperatures can promote the growth, survival, and environmental concentration of *Vibrio cholerae* in aquatic reservoirs. Additionally, periods of high temperature may increase water demand and reliance on potentially contaminated water sources, thereby increasing opportunities for exposure [39]. The observed association is therefore likely driven by a combination of environmental and behavioral factors rather than a single mechanism. Similar delayed effects of temperature on cholera cases have also been reported in Mozambique and the coastline of Kenya [40–42]. On the other hand, a minimum temperature of 15°C was related to an increased RR at longer lags (14 and 28 days), which means that even moderate cooling periods could have an impact in the environment that will eventually lead to cholera transmission, provided that the temperature stays above the minimum threshold necessary for the growth of the pathogen. These results are in line with studies from Tanzania and Vietnam where cool temperatures were found to have a complicated lagged impact on cholera incidence [43,44]. Human infection with cholera has a very short incubation period (from a few hours to 5 days), however, population, level outbreak response to environmental forcing typically shows delays beyond individual incubation because the complexity of exposure pathways requires changes in the environment (bacterial proliferation in reservoirs, contamination of water sources, accumulation and dissemination) and different delays in exposure mechanisms [45–47].

Rainfall was significantly associated with cholera risk in the DLNM analysis. Rainfall was associated with increased cholera risk, consistent with previous evidence that runoff, contamination of water sources, and sanitation failures contribute to transmission [48,49]. Nevertheless, the relationship was not uniform for all years, indicating that other contextual factors such as the provision of drainage facilities, proper sanitation, and flood control measures, may have influenced the association between the two variables. There was also a moderate influence of humidity that was somewhat deferred. The most prominent effect was observed at the humidity level of 60% with the time lag of about 7 days. This similar with the data from the West African region, where it has been reported that due to the high humidity the bacteria *Vibrio cholerae* had a greater chance to survive in surface waters which, in turn, resulted in the continuation of the transmission during the period of humid months [50].

The complex, lagged relationships between climatic variables and cholera risk identified in this study have practical implications. Considering climate variables in early warning systems could make the prediction and preparation for the cholera outbreak more effective by indicating the vulnerable groups for the targeted intervention and the development of early warning systems for the enhancement of the prevention regime [49]. Thus, the public health officials can use temperature and rainfall predictions to plan well, timed interventions that may include, for example, watering quality monitoring, hygiene promotion ahead of the monsoon, and the use of oral cholera vaccines [50].

This study was based on a large, longitudinal dataset; however, there are some limitations, as the environmental data that were used came from satellite sources, which have accuracy limitations and also the datasets are of high spatial resolution; they may not be able to capture local microclimatic variations. In future research, consideration should be given to including neighborhood, level WASH indicators, and also community-level mobility patterns to improve the environmental models.

## Conclusion

This 20-year analysis of laboratory-confirmed cholera in Dhaka demonstrates a clear epidemiological shift, with adults now accounting for the majority of cholera cases, alongside persistent seasonal peaks and strong associations with temperature, rainfall, and humidity. These findings suggest that cholera prevention strategies should move beyond a traditional focus on young children and adopt age-inclusive approaches, including targeted vaccination, enhanced WASH interventions, and climate-informed early warning systems. Integrating environmental surveillance with routine cholera monitoring may improve outbreak prediction and support more effective control efforts in rapidly urbanizing settings.

## Supporting information

S1 Table Distribution of acute watery diarrhea (AWD) cases by age group, year, season, and gender in Dhaka (2004–2024). (DOCX)

S2 Table Seasonal variation in environmental parameters (temperature, humidity, and precipitation) and also AWD and cholera cases in Dhaka (2004–2024). (DOCX)

S1 Fig The simple time series plot cholera with environmental variables. The simple time series plot shows the monthly trends of cholera cases alongside key environmental variables: precipitation, relative humidity, and maximum temperature (Maximum) from April 2003 to August 2025. Cholera cases (red line) appear to show seasonal fluctuations that align closely with peaks in precipitation (cyan line) and increases in temperature (purple line), while relative humidity (yellow line) shows a strong annual cyclic pattern but with less visible synchrony with cholera trends.
(TIF)

S2 Fig Annual relationship between maximum temperature and monthly cholera cases (2004–2024). (TIF)

S3 Fig Annual relationship between minimum temperature and monthly cholera cases (2004–2024). (TIF)

S4 Fig Annual relationship between humidity and monthly cholera cases (2004–2024). (TIF)

S5 Fig Annual relationship between rainfall and monthly cholera cases (2004–2024). (TIF)

S6 Fig Sensitivity analysis of the DLNM results. It varying the maximum lag period (7–28 days), the flexibility of the exposure function (2–5 degrees of freedom), and the assumed count distribution [quasi-Poisson (quasi vs. negative binomial (nb)]. The cumulative relative risks (RRs) at the 95th percentile of exposure was generally consistent across all model specifications.
(TIF)
