# Supplementary material for: Two decades of cholera in Dhaka, Bangladesh: Epidemiology, seasonal patterns, and environmental risk, insights from 2004–2024
Source: PLoS Negl Trop Dis. 2026 Aug 12;20(8):e0014620. doi: 10.1371/journal.pntd.0014620 (PMC13465811; doi:10.1371/journal.pntd.0014620)
Supplement: Supplementary file 1 [file pntd.0014620.s001.docx]

**Supplement table 1: Distribution of acute watery diarrhea (AWD) cases by age group, year, season, and gender in Dhaka (2004–2024)**

| **Characteristic** | **Overall, N = 37,361** | **0-5 years, N = 18,013** | **5-18 years, N = 3,023** | **>18 years, N = 16,325** |
| --- | --- | --- | --- | --- |
| **Acute watery Diarrhea cases** |  |  |  |  |
| Positive | 37,361 (100%) | 18,013 (100%) | 3,023 (100%) | 16,325 (100%) |
| **Year** |  |  |  |  |
| 2004-2008 | 8,330 (22%) | 3,814 (21%) | 965 (32%) | 3,551 (22%) |
| 2009-2013 | 9,330 (25%) | 4,506 (25%) | 758 (25%) | 4,066 (25%) |
| 2014-2018 | 9,361 (25%) | 4,315 (24%) | 618 (20%) | 4,428 (27%) |
| 2019-2024 | 10,340 (28%) | 5,378 (30%) | 682 (23%) | 4,280 (26%) |
| **Season** |  |  |  |  |
| Monsoon | 5,935 (16%) | 2,673 (15%) | 513 (17%) | 2,749 (17%) |
| Other | 9,648 (26%) | 6,040 (34%) | 572 (19%) | 3,036 (19%) |
| Post-monsoon | 6,349 (17%) | 2,524 (14%) | 636 (21%) | 3,189 (20%) |
| Pre-monsoon | 15,429 (41%) | 6,776 (38%) | 1,302 (43%) | 7,351 (45%) |
| **Gender** |  |  |  |  |
| Female | 15,594 (42%) | 7,256 (40%) | 1,195 (40%) | 7,143 (44%) |
| Male | 21,767 (58%) | 10,757 (60%) | 1,828 (60%) | 9,182 (56%) |
| *^1^* n (%) | | | | |
