# Supplementary material for: Two decades of cholera in Dhaka, Bangladesh: Epidemiology, seasonal patterns, and environmental risk, insights from 2004–2024
Source: PLoS Negl Trop Dis. 2026 Aug 12;20(8):e0014620. doi: 10.1371/journal.pntd.0014620 (PMC13465811; doi:10.1371/journal.pntd.0014620)
Supplement: Supplementary file 2 [file pntd.0014620.s002.docx]

**Supplement table 2. Seasonal variation in environmental parameters (temperature, humidity, and precipitation) and also AWD and cholera cases in Dhaka (2004–2024)**

| **Characteristic** | **Monsoon** | **Other** | **Post-monsoon** | **Pre-monsoon** |
| --- | --- | --- | --- | --- |
| **Acute watery Diarrhea cases** |  |  |  |  |
| Positive | 5,935 | 9,648 | 6,349 | 15,429 |
| **Cholera** |  |  |  |  |
| Positive | 1,000 | 847 | 1,363 | 2,931 |
| **Temperature maximum** |  |  |  |  |
| Minimum | 30.70 | 25.10 | 29.26 | 31.42 |
| Maximum | 31.82 | 27.86 | 31.07 | 36.05 |
| Mean | 31.28 | 26.72 | 30.31 | 34.03 |
| SD | 0.32 | 0.82 | 0.41 | 1.10 |
| **Temperature minimum** |  |  |  |  |
| Minimum | 25.8 | 12.7 | 23.2 | 23.4 |
| Maximum | 26.4 | 16.1 | 25.5 | 24.8 |
| Mean | 26.1 | 14.5 | 24.2 | 24.0 |
| SD | 0.2 | 0.8 | 0.5 | 0.4 |
| **Relative humidity** |  |  |  |  |
| Minimum | 87 | 61 | 83 | 61 |
| Maximum | 91 | 77 | 88 | 81 |
| Mean | 89 | 69 | 86 | 69 |
| SD | 1 | 4 | 1 | 5 |
| **Precipitation** |  |  |  |  |
| Minimum | 7.1 | 0.0 | 3.6 | 4.9 |
| Maximum | 26.7 | 1.7 | 15.6 | 15.4 |
| Mean | 12.2 | 0.7 | 7.9 | 7.7 |
| SD | 4.4 | 0.5 | 3.3 | 2.7 |
